# Direct Measurement of Mammographic X-Ray Spectra with a Digital CdTe Detection System

**DOI:** 10.3390/s120608390

**Published:** 2012-06-18

**Authors:** Leonardo Abbene, Gaetano Gerardi, Fabio Principato, Stefano Del Sordo, Giuseppe Raso

**Affiliations:** 1 Dipartimento di Fisica, Università di Palermo,Viale delle Scienze, Edificio 18, Palermo 90128, Italy; E-Mails: gaetano.gerardi@unipa.it (G.G.); fabio.principato@unipa.it (F.P.); giuseppe.raso@unipa.it (G.R.); 2 INAF/IASF Palermo, Via Ugo La Malfa 153, Palermo 90146, Italy; E-Mail: delsordo@ifc.inaf.it

**Keywords:** X-ray spectroscopy, high photon counting rate, CdTe detectors, digital pulse processing, mammography

## Abstract

In this work we present a detection system, based on a CdTe detector and an innovative digital pulse processing (DPP) system, for high-rate X-ray spectroscopy in mammography (1–30 keV). The DPP system performs a height and shape analysis of the detector pulses, sampled and digitized by a 14-bit, 100 MHz ADC. We show the results of the characterization of the detection system both at low and high photon counting rates by using monoenergetic X-ray sources and a nonclinical X-ray tube. The detection system exhibits excellent performance up to 830 kcps with an energy resolution of 4.5% FWHM at 22.1 keV. Direct measurements of clinical molybdenum X-ray spectra were carried out by using a pinhole collimator and a custom alignment device. A comparison with the attenuation curves and the half value layer values, obtained from the measured and simulated spectra, from an ionization chamber and from a solid state dosimeter, also shows the accuracy of the measurements. These results make the proposed detection system a very attractive tool for both laboratory research, calibration of dosimeters and advanced quality controls in mammography.

## 1. Introduction

The spectral distribution of X-ray beams from X-ray tubes is essential for quality control (QC) in mammography, in terms of image quality and patient dose [1–3]. X-ray spectra can be used for accurate estimations of the peak voltage (KVp) of the tubes [4], the energy fluence rate [5], the inherent filtration [6], the beam-hardening artifacts [3] and for the correct implementation of the new dual-energy techniques [7]. By way of example, the peak voltage of a diagnostic X-ray tube should be routinely monitored, since small KVp changes can modify both absorbed dose and image contrast in mammography [4]. With regard to dosimetric investigations, X-ray spectra can be also used to estimate the exposure, the air kerma and the absorbed energy distribution inside a breast tissue or a test phantom [8], overcoming the well known problem of the energy dependence of the response of the dosimeters (solid state detectors and ionization chambers) which are commonly used for the measurements of the absorbed energy distribution. Dosimeter calibrations, which usually involve complicated and time-consuming procedures, are a critical issue for routine investigations.

The spectrum emitted by a mammographic X-ray tube is, typically, obtained by analytical procedures based on semi-empirical models [2,9,10] and Monte Carlo methods [11–13]. In routine quality controls, insufficient information about some characteristic parameters of the X-ray tubes, such as the anode angle, the filters and the exact value of the applied tube voltage, could compromise the precision and the accuracy of the spectra. Of course, measurement of X-ray spectra is the best procedure for accurate quality controls in mammography. Currently, routine measurement of mammographic X-ray spectra is quite uncommon due to the complexity of the measurement procedure. The measurement of mammographic X-ray spectra is a difficult task because of limitations on measurement with high energy resolution at high photon counting rates as well as geometrical restrictions, especially in a hospital environment.

Due to the high photon fluence rate of the beams produced by a standard mammographic X-ray tube (10^6^–10^7^ photons/mm^2^ s at 65 cm from the focal spot), pulse pileup is the major drawback for a detection system (detector and readout electronics). As well known, the main effects of pile-up phenomena on X-ray spectrum measurement are the worsening of the energy resolution (tail pile-up [14]) and the distortion of the photon energy distribution (peak pile-up [14]). The optimum detector for mammographic X-ray spectroscopy should have good energy resolution (<5% FWHM at 22.1 keV), good detection efficiency (>90% up to 40 keV) and a fast readout electronics in order to minimize pile-up distortions without compromise the energy resolution.

With regard to the detectors, the potential benefits of using CdTe and CdZnTe detectors for X-ray spectroscopy are already well known [15–23]. As pointed out in several works [24–30], thin CdTe/CdZnTe detectors (1–2 mm thick) are very appealing for the development of portable detection systems for mammographic X-ray spectroscopy (1–40 keV), ensuring high detection efficiency, good energy resolution, low tailing and no X-ray escape in the measured spectra.

Beside the detector, the readout electronics also plays a key role in the development of a detection system. In a typical readout electronics setup, the detector signals (preamplifier output) are shaped and filtered by an analog shaping amplifier and finally processed by a multichannel analyzer (MCA) to generate the energy spectrum. For high-rate X-ray spectroscopy, the temporal width of the shaped pulses (adjusted by the shaping time constant of the amplifier) and the processing time of the MCA should be as short as possible in order to minimize pile-up effects and counting losses. In the last decade, several groups [26–30] have proposed detection systems, based on CdTe and CdZnTe detectors, wherein the shaped pulses are sampled by a digitizer (with a sampling frequency of 20 MHz) minimizing the processing time of MCAs. The digitized shaped pulses are processed off-line for pulse height analysis and pile-up inspections. These systems show good spectroscopic performance up to a photon counting rate of 50 kcps, limit due to the width of the shaped pulses (660 ns FWHM). Recently, the dramatic performance improvement of the digitizers (with sampling frequencies >100 MHz and a bit number >12) has stimulated an intensive research and development on digital systems, based on digital pulse processing (DPP) techniques, able to directly sample the detector signals and to generate the energy spectra. The DPP techniques allow implementation of complex algorithms, that are not easily implementable through a traditional analog approach, ensuring higher throughput, adaptive processing, flexibility, stability and better pile-up corrections, especially in a high counting rate environment (>50 kcps).

In this work we report on the performance of an X-ray detection system based on a CdTe detector coupled to an innovative digital pulse processing (DPP) system for high-rate X-ray spectroscopy in the mammographic energy range (1–30 keV). This work was carried out in the sequence of previously developed DPP systems [31–33] with the goal of developing a digital spectrometer based on DPP techniques and characterized by high performance both at low and high photon counting rate environments. We investigated on the response of the digital detection system both at low and at high photon counting rates (up to 830 kcps) by using monoenergetic X-ray sources, nonclinical X-ray tubes (Ag, Mo, W, anode materials) and a clinical Mo anode X-ray tube. As an application, precise estimations of the peak voltage of the X-ray tube were also performed. Attenuation curves and half value layer (HVL) values, obtained from the measured and simulated spectra, from an ionization chamber and from a solid state dosimeter, were also compared. In particular, the comparison with the attenuation curve, measured by using the solid state dosimeter, highlighted, first, the energy dependence of the dosimeter response and, second, the importance to measure the energy spectra to avoid the needed calibrations of dosimeters.

## 2. Materials and Methods

### 2.1. Detector

The detector is based on a thin CdTe crystal (2 × 2 × 1 mm^3^), wherein both the anode (indium) and the cathode (platinum) are planar electrodes covering the entire detector surface. The Schottky barrier at the In/CdTe interface ensures low leakage current even at high bias voltage operation (400 V), thus improving the charge collection efficiency. A Peltier cell cools both the CdTe crystal and the input FET of the charge sensitive preamplifier (A250, Amptek, Bedford, MA, USA) at a temperature of −20 °C. The detector cooling reduces the leakage current, allowing the application of higher bias voltages to the electrodes; moreover, the FET cooling increases its transconductance and reduces the electronic noise. All measurements were performed at the temperature of −20 °C. The detector, the FET and the Peltier cooler are mounted in a hermetic package equipped with a light-vacuum tight beryllium window (a modified version of Amptek XR100T-CdTe, S/N 6012). To increase the maximum counting rate of the preamplifier, a feedback resistor of 1 Gω and a feedback capacitor of 0.1 pF were used.

### 2.2. Digital System

The DPP system consists of a digitizer and a PC wherein the digital analysis of the detector pulses was implemented. The detector signals (preamplifier output signals) were directly digitized by using a 14-bit, 100 MHz digitizer (NI5122, National Instruments, Austin, TX, USA). The digital data were acquired and recorded by a Labview program on the PC platform and then processed off-line by a custom digital pulse processing method (C++ coded software) developed by our group. The analysis time is very short, about 3 times the acquisition time. The digital method, combining fast and slow shaping, automatic pole-zero cancellation, baseline restoration, pile-up rejection and pulse shape discrimination, allows precise pulse height measurements even at high counting rates. The digitized pulses were shaped by using the classical single delay line (SDL) shaping technique. Each shaped pulse is achieved by subtracting from the original pulse its delayed and attenuated fraction. This attenuation acts as pole-zero cancellation. The width of each shaped pulse is equal to *T_d_* + *T_p_*, wherein *T_d_* is the delay time and *T_p_* is the peaking time of the related preamplifier output pulse. Our DPP method is characterized by two shaping modes: a “*fast*” SDL shaping mode and a “*slow*” SDL shaping mode. The “*fast*” shaping operation, characterized by a short delay time *T_d,fast_*, is optimized to detect the pulses and to provide a pile-up inspection. If the width of the shaped pulses exceeds a maximum width threshold then the pulse is classified as representative of pile-up events; whenever it is possible, each overlapped event is recognized through a peak detection analysis. Obviously, these events are not analyzed by the “*slow*” shaping procedure. The delay time of the “*fast*” shaping operation is a dead time for the DPP system (paralyzable dead time) and it must be as small as possible, depending of detector and ADC characteristics. In this work we used *T_d,fast_* = 60 ns. The “*slow*” shaping operation, which has a long delay time *T_d,slow_*, is optimized to shorten the pulse width and minimize the ballistic deficit. To obtain a precise pulse height measurement, a convolution of the shaped pulses with a Gaussian function was performed. The slow delay time *T_d,slow_* acts as the shaping time constant of an analog shaping amplifier: the proper choice depends on the peaking time of the preamplifier pulses, the noise and the incoming photon counting rate.

To ensure good energy resolution also at high photon counting rates, a standard detection system is typically equipped with a baseline restorer which minimize the fluctuations of the baseline. The digital method performs a baseline recovery by evaluating the mean value of the samples, within a time window equal to *T_d,slow_*/2, before and after each shaped pulse. This operation sets a minimum time spacing between the pulses equal to *T_a_* = *2*·*T_d,slow_* + *T_p_* for which no mutual interference must exist in the baseline measurement. The minimum time spacing *T_a_* is used to decide whether the events must be discarded, in particular if the time spacing does not exceed *T_a_* the two events are rejected. This operation acts as a pile-up rejector (PUR). It is clear that a *T_d,slow_* value too long reduces the number of the counts in the measured spectrum and its optimum value is the best compromise between the required energy resolution and throughput (*i.e.*, the output photon counting rate). The time *T_a_* is paralyzable dead time for the *slow* shaping operation. In this work we used a *T_d,slow_* = 3 μs.

As shown in our previous works [32,33], we also implemented a pulse shape discrimination (PSD) technique to minimize peak pile-up events, *i.e.*, overlapped preamplified pulses within the peaking time that are not detectable through the “*fast*” shaping operation. This technique is based on the selection of a range of peaking time values of the pulses that are not piled-up.

To perform pulse shape discrimination, we carried out the measurement of the peaking time of the analyzed pulses (after SDL shaping). We first evaluate the rise time of the pulses, *i.e.*, the interval between the times at which the shaped pulse reaches 10% and 90% of its height (after baseline restoration). The times, corresponding to the exact fractions (10% and 90%) of the pulse height, are obtained through a linear interpolation. We estimate the peaking time equal to 2.27 times the rise time (*i.e.*, about five times the time constant). Due to the precise measurement of the pulse height-baseline and interpolation, the method allows fine peaking time estimations (with a precision of 2 ns) both at low and high photon counting rates.

### 2.3. Characterization of the System

To characterize the spectroscopic performance of the system in the mammographic energy range, we measured the response of the system to a ^109^Cd source (22.1 keV, 24.9 keV and 88.1 keV) at low (200 cps) and high (830 kcps) photon counting rates. The energy calibration of the detection system was obtained by using further calibration sources (^241^Am: 59.5 keV; ^57^Co: 122.1, 136.5 keV) and the linearity of the digital system was also verified. The measured spectra were analyzed by using a custom function model, which takes into account both the symmetric and the asymmetric peak distortion effects [17]. Statistical errors on the spectroscopic parameters with a confidence level of 68% were associated. We also performed measurements of non clinical X-ray spectra at the “Livio Scarsi” Laboratory (LAX) [34], located at DIFI (Palermo, Italy). The facility is able to produce X-ray beams with an operational energy range of 0.1–60 keV (tube anodes: Ag, Co, Cr, Cu, Fe, Mo, W), collimated on a length of 10.5 m with a diameter at full aperture of 200 mm. In this work, we used W and Ag targets.

### 2.4. Peak Voltage Measurements

Precision and accuracy in the estimation of the peak voltage of X-ray tubes are crucial in mammography. Small changes in peak voltages can produce significant modifications of the absorbed dose and image contrast. As proposed in several works [4,35,36], it is possible to perform precise estimations of peak voltage through the end point energy of the measured spectra (non-invasive measurements). The tube voltage is obtained through the intersection point (in keV) of two straight lines, one for the background beyond the end point energy and the other for the final region of the spectrum. The two lines were evaluated by a linear best fit procedure of the experimental points. Uncertainties evaluated for the voltage values are less than 0.22% (confidence level of 68%) for voltages in the range 28–32 kV.

### 2.5. Clinical Measurements

**Figure 1.**
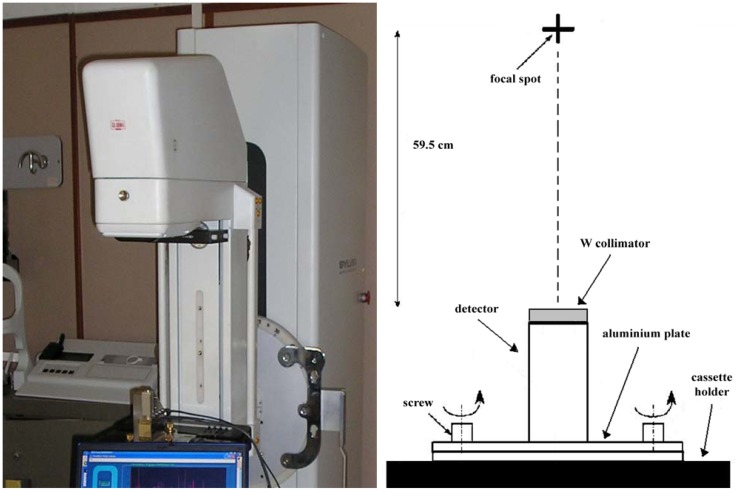
Experimental set-up of clinical measurements of mammographic X-ray spectra with the digital system.

Mo-target X-ray spectrum measurements were performed under clinical conditions (Istituto di Radiologia, Policlinico, Palermo). We used a Sylvia mammographic unit (Gilardoni, Mandello del Lario, Italy) with a Mo anode tube (MCS, 50MOH), characterized by an additional filtration of 0.03 mm Mo and a 10° anode angle. The compression paddle was removed during the measurements. The detector was placed on the cassette holder with a 59.5 cm focal spot-detector distance (Figure 1).

To reduce the photon counting rate on the detection system to an acceptable level, we used a pinhole collimator: a tungsten collimator disk, 1 mm thick with a 100 μm diameter circular hole, placed in front of the detector (over the beryllium window). Using this collimator setup, we measured X-ray spectra with a photon counting rate up to 450 kcps. It is well known that the choice of the proper collimation system is a critical issue for accurate measurements of X-ray spectra. An excessive reduction of the aperture and the thickness of the collimator can produce several distortions in the measured spectra (peaks and continuum events). These distortions, as well described by several authors [37,38], are mainly due to: (i) the penetration of photons through the collimator material; (ii) scattered photons from the collimator edges; and (iii) characteristic X rays from the collimator material. The first effect can be reduced by choosing a proper collimator material and thickness. In the investigated energy range (1–30 keV), the penetration of photons through the 1 mm thick tungsten collimator is negligible, as demonstrated by the estimated values of the transmission equivalent aperture (*TEA*, [38]). By using the tabulated tungsten mass attenuation coefficient values [39], we obtained a *TEA* equals to the collimator aperture area, showing that no photon penetration occurs.

The other collimation distortions mainly depend on the alignment of the collimator with the X-ray beam and on the energy of the X-ray beam. Misalignment between the X-ray beam and the collimator can produce scattered photons and characteristic X rays from the collimator edge. Obviously, accurate alignment becomes more difficult as the thickness of the collimator increases and its aperture diameter decreases. The optimum collimator set-up should be a trade-off between the reduction of collimation distortions and the photon counting rate. To optimize the beam-detector alignment, the detector was mounted on an aluminum plate equipped with three micrometric screws. A preliminary focal spot-detector alignment was carried out with a laser pointer taking into account the reference marks positioned on both sides of the tube head, while a more accurate alignment was obtained by changing the plate orientation looking for the maximum photon counting rate and the absence of distortions in the measured spectra.

We also performed the measurement of attenuation curves and the half value layer (HVL) estimation through the measured Mo spectra. The estimated HVL value (*i.e.*, the thickness of some standard material required to reduce the exposure of a beam to half its original value), which is usually measured to characterize the spectral properties of the beams, was compared with that measured by using a standard mammographic ionization chamber (Magna 1cc together with Solidose 400, RTI Electronics, Mölndal, Sweden) and a solid state dosimeter (R100, RTI Electronics). Exposure values from spectral data were obtained through the estimation of the energy fluence and the air mass energy absorption coefficients, as described in our previous work [28]. To measure the attenuation curves and the HVL values, we used a standard aluminum filter set (type 1100, Al 99.0% purity, RTI electronics). To minimize the effects of scattered radiation, we performed the HVL measurements in a “good geometry” condition, as suggested by several authors [40–42]; in particular, the experimental set-up for HVL measurements was characterized by a filter-detector distance of 42 cm. The HVL values were obtained from the attenuation curves by interpolation of the two data points neighbouring the HVL thickness [43]. Errors with a confidence level of 68% were associated on the overall measured parameters.

## 3. Results and Discussion

### 3.1. Response of the System

**Figure 2.**
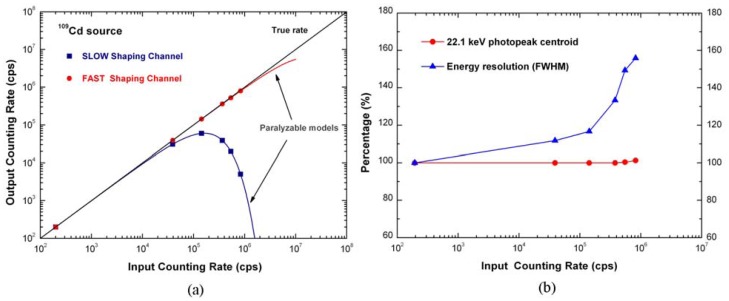
(**a**) Throughput of the system: the measured photon counting rates from “fast” and “slow” channels (red and blue points, respectively) versus the true input counting rate. The red and blue lines are the paralyzable dead time model functions for the “fast” (dead time of 60 ns) and “slow” (dead time of 6.14 μs) channels, respectively; (**b**) The 22.1 keV photopeak centroid and energy resolution (FWHM) at 22.1 keV (percent deviation from the values at 200 cps).

Figure 2 shows the performance of the detection system, irradiated with the ^109^Cd source, at different photon counting rates (up to 830 kcps). We reported the rate of the events analyzed by the “*fast*” and the “*slow*” pulse shaping channels versus the true input counting rate (Figure 1(a)). The true input counting rate was estimated from the fast channel rate by using the paralyzable dead time model (dead time of 60 ns) [14]. As shown in Figure 1(a), both the “*fast*” and the “*slow*” rates are in agreement with the paralyzable dead time model [14]. Therefore, despite an high throughput reduction, due to the PUR operation, it is always possible to estimate the true rate of the impinging photons through the “fast” channel. Figure 2(b) shows the 22.1 keV photopeak centroid and the energy resolution (FWHM) at 22.1 keV (percent deviation from the values at 200 cps), measured at different photon counting rates. The photopeak centroid shift was less than 1% and low energy resolution worsening characterized the system. The system, through both “slow” and “fast” channels is able to determine the input count rate and the energy spectrum with high accuracy and precision even at high photon counting rates.

**Figure 3.**
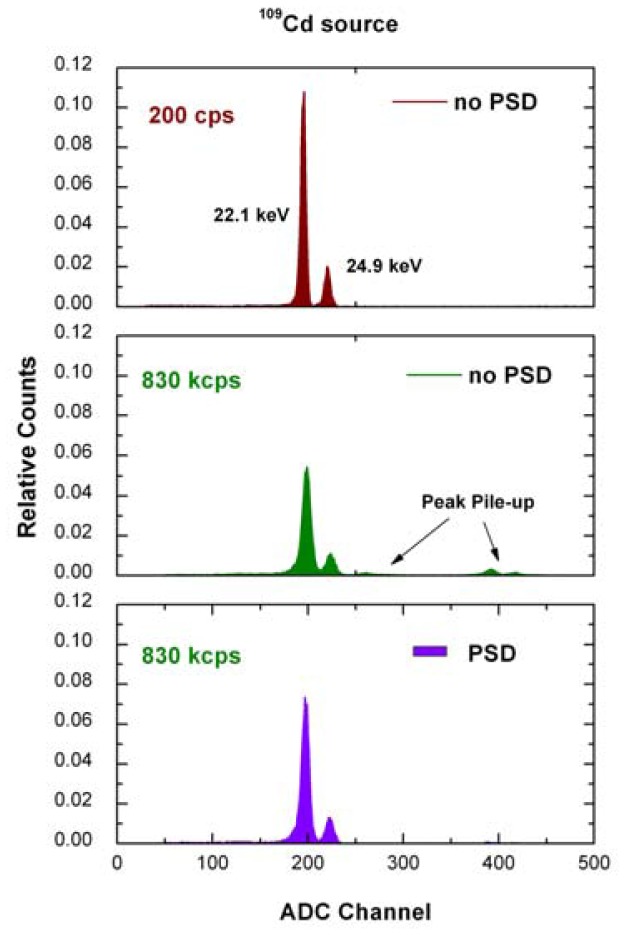
Measured ^109^Cd spectra at 200 cps with no correction, at 830 kcps with no correction and at 830 kcps after PSD. The counts were normalized to the total number of detected events.

Figure 3 shows the measured ^109^Cd spectra at 200 cps and 830 kcps. Low tailing characterizes the full-energy peaks of the spectra and no escape peaks are visible, confirming the appealing features of CdTe detectors in the mammographic energy range. Moreover, it is clearly visible the strong reduction (96%) of the number of peak pile-up events in the measured spectrum by using the pulse shape discrimination (PSD) technique (pulses with a peaking time more than 148 ns were discarded). Table 1 summarizes the spectroscopic results (energy resolution and photon counting rate).

**Figure 4.**
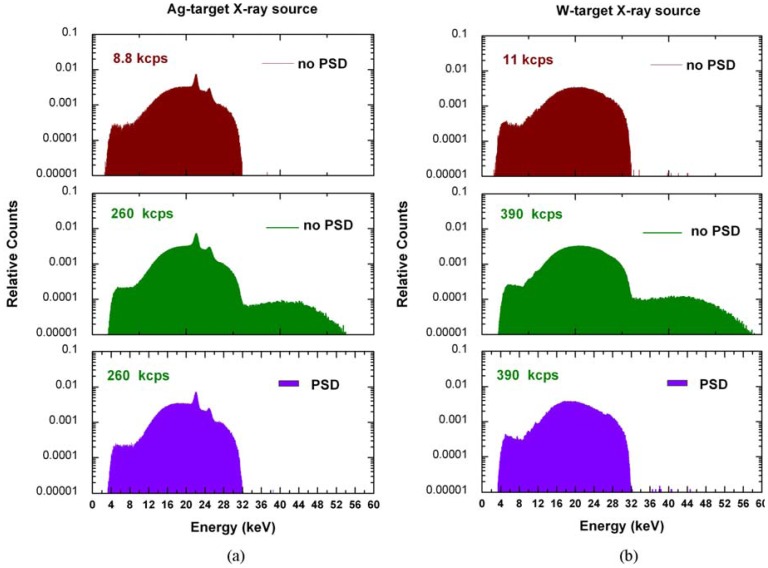
Measured Ag- and W-target X-ray spectra (32 kV) with no correction and after PSD. The counts were normalized to the total number of detected events.

We also measured X-ray spectra from a non clinical X-ray tube with different anode materials (Ag and W). In Figure 4(a) are shown the measured Ag-target X-ray spectra (32 kV) at 8.8 kcps with no correction, at 260 kcps with no correction and at 260 kcps after PSD. At high photon counting rate, the measured Ag spectrum, despite the good energy resolution of the peaks (22.1 and 24.9 keV), is characterized by a high background beyond the end point energy, due to the peak pile-up; while, after PSD, this background is quite similar to the spectrum at low photon counting rate.

**Figure 5.**
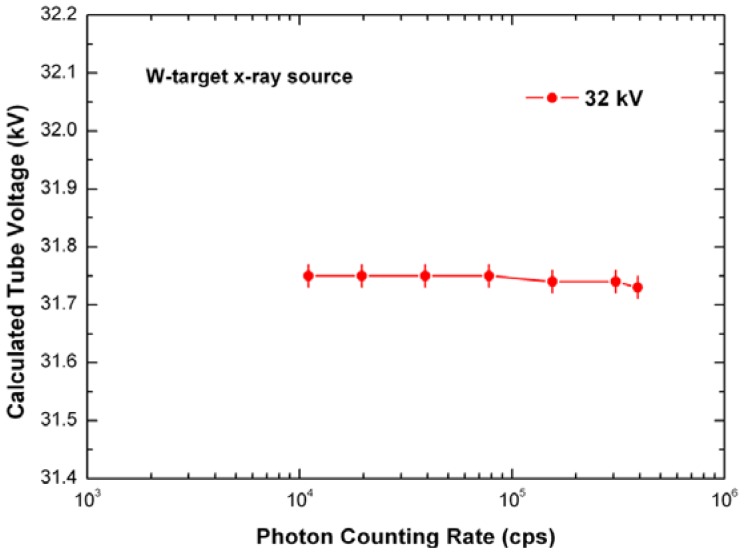
Calculated tube voltage values *vs.* photon counting rate (W anode). Relative errors less than 0.22%, with a confidence level of 68%.

These results open up the possibility of precise estimations of the end point energy, *i.e.*, the peak voltage of a X-ray tube, even at high photon counting rates. Figure 4(b) also shows the measured W-target X-ray spectra. As an application, we measured the peak voltage of the X-ray tube, as described in section 2.4, at 32 kV and at different photon counting rates (up to 390 kcps, W anode). Figure 5 shows no significative variation of the calculated tube voltage values (relative errors less than 0.22%, with confidence level of 68%) up to 390 kcps. This result points out the ability of the system to perform peak voltage measurements with high precision even at high rates, contrary to what happen using standard systems [36].

### 3.2. Clinical X-Ray Spectroscopy

**Figure 6.**
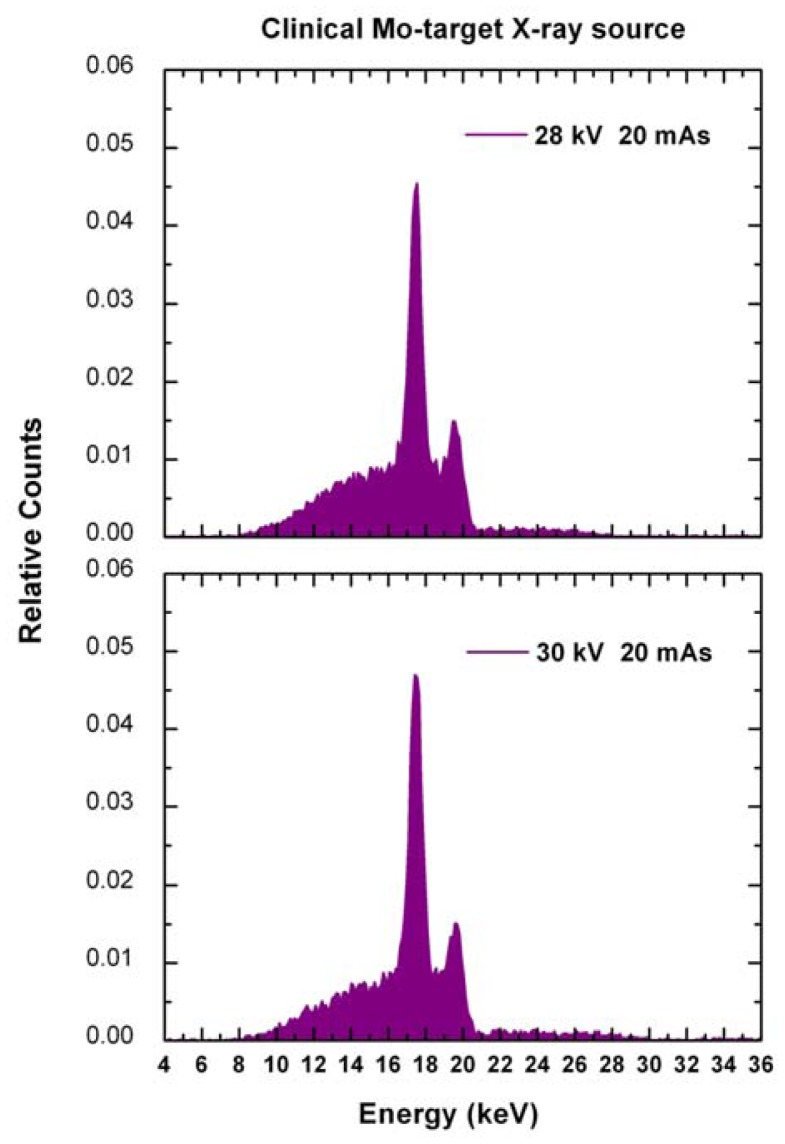
The Mo-target X-ray spectra measured with the digital system under clinical conditions (28 kV and 30 kV, 20 mAs). The counts were normalized to the total number of detected events.

**Figure 7.**
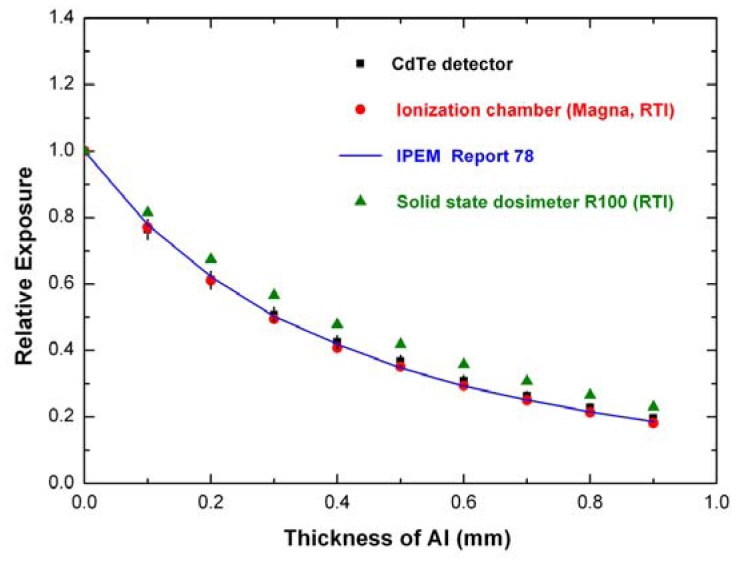
Attenuation curves obtained from measured and simulated spectra and from direct exposure measurements (ionization chamber and solid state detector). The tube settings were: 28 kV and 20 mAs.

Figure 6 shows the measured Mo-target X-ray spectra under clinical conditions. The tube settings were: tube voltages of 28 and 30 kV and tube current-time product of 20 mAs. The photon counting rates are 360 kcps and 450 kcps at 28 kV and 30 kV, respectively. Figure 7 shows the attenuation curves (28 kV and 20 mAs) obtained from the spectra measured with the digital system, from simulated spectra (IPEM Report 78) and by using the exposure values directly measured with the ionization chamber (Magna 1cc together with Solidose 400, RTI Electronics) and with the solid state dosimeter (R100, RTI Electronics). It is well evident the good agreement among the curves and the HVL values (Table 2), obtained from the detector, the simulation and the ionization chamber. The HVL errors, with a confidence level of 68%, were calculated by using the method suggested by Wagner [43]. The agreement between the measured HVL values with the standard value (standard radiation qualities RQR-M 2 [42]) also points out the “good geometry” condition of the experimental set-up for HVL measurements. The disagreement with the attenuation curve and the HVL value (Table 2), obtained from the solid state dosimeter, points out the energy dependence of the dosimeter response. Since aluminum filters harden the X-ray beam and alter the energy spectrum, if dosimeter does not has a *flat* response for different spectra, the HVL measurement will be in error. The correction of the energy dependence of the dosimeter response need accurate calibrations which involve complicated and time-consuming procedures, critical for routine investigations.

These comparisons highlight two main aspects: (i) the ability of the digital system to perform accurate mammographic X-ray spectra without excessive time-consuming procedures; and (ii) the possible use of this system both as dosimeter and for calibrations of dosimeters.

## 4. Conclusions

The performance of a detection system for X-ray spectroscopy in the mammographic energy range (1–30 keV) was presented. The system, based on a CdTe detector and on an innovative digital pulse processing (DPP) system, shows excellent spectroscopic performance, even at high photon counting rates (energy resolution of 4.5% FWHM at 22.1 keV at 830 kcps). Beside the appealing characteristics of the CdTe detector (good energy resolution, high detection efficiency, response), these excellent results are mainly due to the high-rate capability of the DPP system. This system, developed by our group, performs the typical pulse processing techniques—filtering, pile-up inspection, baseline restoration, pulse height measurement and pulse shape discrimination—traditionally implemented through analog circuits. This new approach, through the direct sampling of the detector signals and a digital analysis, gives better results than the traditional analog systems. Measurements of clinical and non clinical X-ray spectra highlight the high potentialities of the detection system for X-ray spectroscopy in mammography. The high rate capability of the system allows to use larger collimators (100 μm diameter circular hole) than those typically used for mammographic X-ray spectroscopy (50 μm, 25 μm), minimizing the collimator distortions in the measured spectra (characteristic peaks and scattered events) and the beam/detector alignment time which is a critical issue for routine applications. Moreover, the possibility of using large collimators also minimizes any conicity (more critical for small collimators) allowing precise estimations of the photon fluence.

The ability of the digital system to perform accurate X-ray spectrum measurements, even at high photon counting rates, is also helpful: (i) to perform precise estimations of the peak voltage of the X-ray tubes; and (ii) to overcome the energy dependence of the response of the dosimeters, wherein changes in the shape of the spectra can severely alter the estimation of the dose.

The results open up the development of new detection systems for spectral X-ray imaging in mammography, based on CdTe/CdZnTe pixel detectors coupled with a DPP system. Recently, single photon counting detectors are very appealing for digital mammography allowing the implementation of dual-energy techniques and improvements on the quality of images [44]. In this contest, CdTe/CdZnTe pixel detectors [45–48] could to ensure better performance (energy resolution <5% at 30 keV) than the current prototypes based on silicon detectors (energy resolution of about 15% at 30 keV [44]).

Further tests will concern the use of the DPP system, coupled to a CdTe/CdZnTe detector with higher detection efficiency, for X-ray spectrum measurements in computed tomography (20–140 keV). Currently, we are involved in the development of a real time system, based on the digital method, by using a field programmable gate array (FPGA) technology. Both the acquisition (with no dead time) and the analysis have been successfully simulated.

## Figures and Tables

**Table 1.** Spectroscopic results at 22.1 keV.

	**No correction**	**PSD**
Energy resolution *FWHM* (%)	4.77 ± 0.04	4.52 ± 0.04
True input rate (kcps)	830	830
Measured output rate (kcps)	5.1	1.3

**Table 2.** HVL measurements.

	**HVL (mm Al)**
Measured with the CdTe detector	0.31 ± 0.01
Measured with the ionization chamber	0.300 ± 0.009
Measured with solid state dosimeter	0.380 ± 0.009
Calculated from IPEM Report 78	0.311
Radiation quality RQR-M 2	0.310
